# Environmental Performance of 3D-Formed Recycled Fiber-Reinforced Foamed Concrete: Leaching, Thermal Stability, and Microbiological Assessment

**DOI:** 10.3390/ma19173647

**Published:** 2026-08-27

**Authors:** Magdalena Rudziewicz, Magdalena Szechyńska-Hebda, Marek Hebda

**Affiliations:** 1 Faculty of Materials Engineering and Physics, Cracow University of Technology, Warszawska 24, 31-155 Krakow, Poland; magdalena.rudziewicz@doktorant.pk.edu.pl; 2 W. Szafer Institute of Botany Polish Academy of Sciences, Lubicz 46, 31-512 Krakow, Poland; m.szechynska-hebda@botany.pl; 3 Faculty of Mechanical Engineering, Cracow University of Technology, Warszawska 24, 31-155 Krakow, Poland

**Keywords:** 3D-printed cementitious composites, alkali-activated materials, thermal stability, microbial resistance

## Abstract

The increasing adoption of additive manufacturing in the construction sector has intensified the demand for lightweight, recyclable cement-based composites with low environmental impact, suitable for automated 3D printing. Foamed concrete reinforced with dispersed fibers and incorporating recycled constituents represents a promising class of multifunctional materials. However, its environmental performance remains insufficiently characterized. This study provides a comprehensive evaluation of thermal stability, leaching behavior, and microbial resistance of 3D-printable fiber-reinforced foamed cement composites produced with recycled components. Thermogravimetric–Fourier transform infrared (TG–FTIR) analysis confirmed a characteristic three-stage thermal decomposition pathway typical of hydrated cementitious systems. All composites exhibited high thermal stability, with residual masses of 88.56–90.17% at 900 °C. The binder type exerted a stronger influence on decomposition behavior than atmospheric exposure or freeze–thaw conditioning. Leaching tests revealed strongly alkaline eluates (pH 11.0–11.4), low total organic carbon (<0.6 wt.%), and only trace concentrations of BTEX (35–41 μg/kg), PAHs, and PCBs. Alkali activation increased the release of chromium (3.6–4.0 mg/kg), arsenic (1.2 mg/kg), copper (4.5 mg/kg), antimony (0.26 mg/kg), and sulfates (2700–4700 mg/kg), accompanied by elevated total dissolved solids (~18,000 mg/kg). Nevertheless, all environmentally relevant constituents remained well below the waste acceptance criteria (WAC), confirming effective immobilization of hazardous species within the hardened matrix. The results provide new insights into the relationships among material composition, porous microstructure, and environmental safety, demonstrating that the developed 3D-printable foamed composites exhibit robust performance, suitability for safe and durable applications, and favorable environmental performance.

## 1. Introduction

Additive manufacturing technologies are increasingly being implemented in the construction sector, enabling the production of complex components with reduced material consumption, shorter construction times, and lower labor requirements [1,2]. Among these technologies, 3D printing of cement-based materials has attracted particular attention as a promising approach for sustainable and automated construction processes [3,4,5]. The development of printable cement-based composites requires not only appropriate rheological and mechanical properties [6,7] but also environmental and health safety throughout the entire material life cycle [8,9]. Foamed concrete has emerged as an attractive material for additive manufacturing due to its low density, thermal insulation properties, and reduced demand for raw materials. Its cellular structure enhances energy efficiency and reduces the overall environmental impact of building components [10]. However, the reduction in density may adversely affect mechanical properties; therefore, dispersed fiber reinforcement is increasingly used to enhance tensile strength, crack resistance, and overall structural integrity [11,12]. Consequently, fiber-reinforced foamed concrete can be considered a new class of multifunctional composites, combining low weight with adequate durability and mechanical stability. Building on previous studies conducted by the authors, the investigated 3D-printed fiber-reinforced foamed concrete incorporating recycled constituents has demonstrated promising mechanical performance and potential for further development [13,14]. However, beyond mechanical characterization, it is essential to evaluate whether such materials also satisfy environmental and health-related safety requirements before their broader implementation in construction. Environmental safety should therefore be considered as a multidimensional concept encompassing the potential release of hazardous substances, thermal stability, biological durability, and the long-term interaction of construction materials with their surrounding environment. A comprehensive assessment of these aspects is essential for ensuring the sustainable application of novel cement-based composites. At the same time, the construction sector is undergoing a transition toward circular economy principles, in which secondary and recycled materials are increasingly incorporated into building products [15,16]. Although the incorporation of recycled constituents into cement-based composites contributes to waste reduction and the conservation of natural resources, it also raises concerns regarding the potential release of hazardous substances and environmentally relevant trace elements, as well as the long-term environmental safety of such materials [17,18,19].

The investigated composites incorporate several recycled constituents that contribute to resource efficiency and support circular economy strategies by replacing virgin raw materials and valorizing industrial or post-consumer waste streams. The expected environmental benefits associated with the individual recycled components are summarized in Table 1.

Although a quantitative Life Cycle Assessment (LCA) was beyond the scope of the present study, previous studies have demonstrated that the replacement of virgin raw materials with recycled alternatives may contribute to reductions in embodied carbon, energy demand, and landfill disposal.

Recycled constituents may influence not only the mechanical properties of cement-based composites but also their thermal stability and degradation behavior [24,25]. During exposure to elevated temperatures, hydrated cementitious materials undergo a series of physicochemical transformations associated with the dehydration of hydration products, dehydroxylation reactions, and carbonate decomposition. These processes may affect the structural integrity of the material and are accompanied by the evolution of gaseous products originating from both the cement matrix and incorporated recycled constituents [26]. Consequently, thermal analysis provides valuable information on decomposition mechanisms, characteristic transformation temperatures, and gas evolution during heating, contributing to a more comprehensive evaluation of material performance under fire exposure, high-temperature service conditions, and end-of-life thermal treatment [27,28,29]. Previous studies have demonstrated that recycled fibers, industrial by-products, and other secondary raw materials may modify both the stability of hydration products and the sequence of thermal decomposition reactions, highlighting the importance of thermal characterization in environmentally oriented material design [30,31].

One of the key aspects of environmental safety is the release of chemical constituents from building materials when exposed to water or moisture. During their service life, cement-based composites may be subjected to precipitation, groundwater infiltration, cyclic wetting–drying, or high-humidity conditions, all of which promote the dissolution and transport of soluble constituents from the cementitious matrix. The extent of leaching depends on the physicochemical properties of the material and environmental conditions and may continue over several decades of service life. Among the elements of greatest environmental concern are chromium (particularly Cr(VI)), lead, cadmium, nickel, copper, and zinc, which may be released in trace concentrations and subsequently migrate into surrounding soils or groundwater. Although the measured concentrations are typically within the range of a few μg L^−1^ to several mg L^−1^, long-term cumulative release may become environmentally significant, particularly for construction materials containing industrial by-products or recycled constituents [32,33,34].

The environmental performance of cement-based materials is strongly influenced by their ability to immobilize potentially hazardous elements within the hydration products [35]. This immobilization is primarily associated with calcium silicate hydrate (C–S–H) and other hydration phases, which retain contaminants through physical encapsulation, adsorption, ion exchange, and chemical incorporation [36,37]. However, long-term environmental exposure, including carbonation and weathering, may gradually alter these binding mechanisms and increase the mobility of selected elements. Consequently, evaluating the leaching behavior of cementitious composites containing recycled constituents remains an important aspect of their environmental assessment [38,39,40].

The leaching behavior of cement-based materials is governed by numerous interacting factors, including the chemical composition of the binder, water-to-binder ratio, pore structure, permeability, degree of hydration, carbonation depth, and the type, particle size, and replacement level of recycled constituents. Previous investigations have demonstrated that supplementary cementitious materials such as fly ash, blast furnace slag, silica fume, and calcined clays may either reduce or increase the release of specific elements depending on their mineralogical composition and their influence on pore refinement and contaminant binding mechanisms. Similarly, recycled concrete aggregates and other secondary raw materials may introduce additional trace elements while simultaneously modifying the transport properties of the cement matrix [41,42,43]. In addition to chemical stability, the interaction between cement-based materials and moisture has another important consequence from the environmental safety perspective. The presence of moisture not only affects contaminant transport but also creates favorable conditions for the development of resident microbial communities.

However, elevated humidity levels combined with a porous microstructure may promote fungal growth, leading to surface degradation, indoor air contamination, and potential health risks [44,45]. Therefore, the resistance of building materials to the growth of material-associated microorganisms is of critical importance [46].

Fungi are among the most common microorganisms colonizing building materials. Their growth may contribute to indoor air contamination through the release of spores, allergens, toxins, and microbial volatile organic compounds, and long-term exposure has been associated with allergic reactions, respiratory diseases, skin irritation, and other adverse health effects [47]. Therefore, the assessment of fungal development on building materials is currently regarded as an important component of indoor environmental quality evaluation. Cement-based materials are characterized by a well-developed porous structure and a rough surface texture, both of which promote moisture retention and the accumulation of contaminants that may serve as nutrient sources for microorganisms [48]. It has been demonstrated that the most important factors governing fungal growth include water activity, chemical composition of the material, pH, temperature, and surface characteristics, particularly porosity and roughness [49]. In porous materials, the ability to absorb and retain moisture plays a key role in the initiation and maintenance of fungal growth, as it provides favorable conditions for spore germination and hyphal development [50,51]. Accordingly, evaluating the susceptibility of newly developed cementitious composites to the growth of material-associated microorganisms constitutes an important aspect of their environmental characterization. In the present study, the microbiological investigations were intended as a preliminary qualitative assessment of resident microbial communities rather than a comprehensive quantitative evaluation of microbial activity. Despite the growing number of studies on 3D-printed cementitious materials, previous research has focused predominantly on rheological performance, printability, and mechanical properties. Considerably less attention has been devoted to the combined assessment of thermal degradation, contaminant leaching, and biological susceptibility, particularly for lightweight foamed composites incorporating recycled constituents. Therefore, the objective of this study was to comprehensively evaluate the environmental performance of representative 3D-printed fiber-reinforced foamed cement composites using complementary TG–DTG–FTIR analysis, standardized leaching tests, and preliminary microbiological investigations. It was hypothesized that, under otherwise identical matrix compositions, the binder system would be the primary factor governing the thermal and environmental performance of the investigated composites, whereas environmental conditioning would have a comparatively smaller effect.

## 2. Materials and Methods

### 2.1. Material Description

The investigated material was a 3D-printed fiber-reinforced foamed concrete incorporating recycled constituents. The composite was developed as a lightweight printable cement-based material for additive manufacturing and constituted the final optimized mixture obtained during the authors’ previous research [52]. The optimization process focused on achieving adequate printability, buildability, mechanical performance, and reduced environmental impact through the incorporation of recycled materials.

The investigated composite was produced using low-emission pozzolanic cement CEM IV/B(V) 42.5 N (ECOPlanet 4B, Holcim, Małgoszcz, Poland) as the primary binder, selected for its reduced carbon footprint and partial incorporation of recycled raw materials. The mixture also contained quartz sand together with secondary mineral constituents, including ground demolition brick (GB), fly ash (FA), and coal slag (SL), which were incorporated to reduce the consumption of virgin resources and promote the valorization of industrial and construction waste. Reinforcement was provided by glass fibers (GF) and post-industrial merino wool fibers (MF), while a protein-based foaming agent was used to generate the cellular structure of the composite. The information is summarized in Table 2. All investigated mixtures were prepared with a constant water-to-solid ratio (w/s) of 0.34 and the same base composition consisting of 27 wt.% cement, 37 wt.% quartz sand, 5 wt.% fly ash, 15 wt.% coal slag, and 15 wt.% ground brick waste. The alkali-activated mixtures were prepared using a 10 M sodium hydroxide solution and sodium silicate mixed at a molar ratio of 2.5, producing an activating solution with a density of 1.45 g/cm^3^, while a synthetic foaming agent (Pianotwór, AS, MEEX AG, Chrzanów, Poland) diluted in a 1:1 ratio was used for foam generation. All mixtures were reinforced with 6 mm-long fibers; the fiber content was calculated as a percentage of the total dry mixture mass; the specimens were cured for six months prior to testing; and all samples were manufactured by 3D printing under the process parameters described in Table 3.

The detailed quantitative mixture composition, optimization procedure, fresh-state properties, 3D-printing process, and mechanical characterization of the investigated composites have been comprehensively reported in the authors’ previous publications [53,54]. The same optimized materials were used throughout the present study without any modification to their composition or manufacturing procedure. Therefore, only information relevant to the current experimental program is presented herein, while the present work focuses exclusively on evaluating the environmental and health-related performance of the developed composites. Particular attention was devoted to assessing the safety of the developed composite from complementary perspectives, including its thermal decomposition behavior, leaching characteristics, and growth of material-associated microorganisms under favorable conditions. These aspects are particularly relevant for lightweight foamed cementitious materials incorporating recycled constituents, whose porous microstructure may influence the transport of moisture, dissolved substances, and gaseous decomposition products.

All specimens used in this study were produced during the same manufacturing campaign using the optimized mixture and identical printing parameters. Depending on the requirements of the individual experimental techniques, the specimens were appropriately prepared prior to analysis.

This study is based on four previously developed mixtures (1-M1, 2-M3, 3-M4, and 4-M6), originally reported in our previous work. Mixtures M1 and M3 are cement-based composites, whereas 3-M4 and 4-M6 are their alkali-activated counterparts. Within each binder system, the mixtures differ only in the proportion of glass fibers (GF) and merino fibers (MF), while the total fiber content was maintained at 0.5 wt.%. Their designation and the experimental program are summarized in Table 4. The selection of specimens for the subsequent stages of the study was based on the findings of the previous investigation and tailored to the objective of each experimental method. Since TG-FTIR analysis and leaching tests primarily characterize the phase composition, hydration products, and chemical stability of the binder matrix, mixtures 2-M3 and 4-M6 were selected for these analyses. These mixtures have identical fiber compositions (0.25 wt.% GF and 0.25 wt.% MF) but differ in the type of binder, allowing the effect of alkaline activation to be evaluated while minimizing the influence of other compositional variables. Accordingly, TG-FTIR analysis was performed on reference (R), atmospheric-exposed (A, six months), and freeze–thaw-exposed (FT) specimens of mixtures 2-M3 and 4-M6. Leaching tests were carried out on the 2-M3 A and 4-M6 A specimens after six months of natural atmospheric exposure.

A different specimen selection strategy was adopted for the microbiological investigations. The susceptibility of cementitious composites to the development of resident microbial communities may depend not only on the type of binder but also on surface characteristics, pore structure, and fiber composition. Therefore, atmospheric-exposed (A) specimens of all four mixtures (1-M1 A, 2-M3 A, 3-M4 A, and 4-M6 A) were included in the microbiological study to provide a comprehensive assessment of their resistance to biological growth under outdoor conditions, as summarized in Table 4.

The experimental procedures summarized in Table 5 have been described in detail in the authors’ previous publications, which also present the mechanical properties of the investigated materials. Therefore, only the key experimental parameters relevant to the present study are included here to improve the reproducibility of the environmental assessment.

### 2.2. Methods

#### 2.2.1. Thermogravimetric Analysis (TG-FTIR)

Thermogravimetric analysis coupled with Fourier-Transform Infrared Spectroscopy (TG-FTIR) was performed to evaluate the thermal stability of the developed composite and to identify gaseous products released during thermal decomposition. The combination of thermogravimetry with evolved gas analysis enables simultaneous determination of mass loss and qualitative identification of volatile species generated during heating, providing valuable insight into the degradation mechanisms of cement-based composites containing recycled constituents. Prior to testing, representative specimens were oven-dried to a constant mass and ground into fine powder. Approximately 40 mg of each sample was placed in an open alumina crucible (Al_2_O_3_, 85 μL) and analyzed using a NETZSCH TG 209 F1 Libra thermogravimetric analyzer (Selb, Germany) coupled with a Fourier Transform Infrared (FTIR) spectrometer. The measurements were carried out in an air atmosphere over a temperature range of 25–900 °C at a constant heating rate of 10 °C/min. The oxidizing atmosphere was selected to reproduce the thermal exposure conditions encountered in service and to ensure complete oxidation of the organic constituents present in the composites, including merino wool fibers and the protein-based foaming agent. All TG–FTIR measurements were performed in triplicate to ensure the repeatability of the experimental results. The characteristic temperatures of the thermal decomposition processes were determined using the onset method and compared with the maxima of the corresponding DTG peaks. The boundaries of the individual decomposition stages were established based on changes in the TG and DTG curves, and the mass losses associated with each stage were calculated from the corresponding TG intervals. The TG curves were used to determine the characteristic mass-loss stages associated with moisture evaporation, dehydration and decomposition of hydration products, degradation of organic constituents, and decarbonation of carbonate phases, whereas the simultaneously recorded FTIR spectra enabled the qualitative identification of the gaseous products evolved during thermal decomposition. The experimental repeatability of the thermogravimetric measurements was within ±0.25–0.45 wt.% for mass loss, which is consistent with the repeatability typically reported for thermogravimetric analyses of cementitious and composite materials.

#### 2.2.2. Leaching Test

The environmental performance of the developed composites was evaluated by batch leaching tests. Batch leaching tests were carried out by an accredited laboratory in accordance with the PN EN 12457-3 standard (two-stage compliance batch leaching test), which specifies the preparation of eluates for the determination of the leaching behavior of granular waste materials and sludges [55]. Prior to testing, the specimens were crushed to a particle size of 0–4 mm and mixed with deionized water in high-density polyethylene (HDPE) bottles using a liquid-to-solid (L/S) ratio of 10 L/kg. The suspensions were agitated for 24 h at 21 ± 2 °C under controlled laboratory conditions, after which the eluates were separated and subjected to physicochemical analyses. The elemental composition of the eluates was determined by inductively coupled plasma optical emission spectrometry (ICP-OES). Chloride ions were measured using an ion-selective electrode (ISE), while ammonium nitrogen was determined with a discrete analyzer. The pH of the eluates was measured using a calibrated electrochemical probe. Total organic carbon (TOC) and dissolved organic carbon (DOC) were determined using a TOC analyzer. Volatile organic compounds (VOCs) were analyzed by gas chromatography–mass spectrometry (GC–MS), whereas highly volatile compounds were determined using headspace gas chromatography–mass spectrometry (HS–GC/MS). In addition, elemental analysis (EA) was performed to determine the elemental composition of the investigated materials.

The analyses were carried out by the accredited laboratory Normec i2 Analytical (Ruda Śląska, Poland), accredited by UKAS. The eluates were analyzed for the principal environmental indicators, including pH, dissolved organic carbon (DOC), total dissolved solids (TDS), chlorides, fluorides, sulfates, and the leachability of selected trace elements, namely As, Ba, Cd, Cr, Cu, Hg, Mo, Ni, Pb, Sb, Se, and Zn. In addition, the acid neutralization capacity (ANC) of the material was determined to assess its buffering potential under acidic environmental conditions. All analyses were performed using accredited analytical procedures, with trace elements determined by inductively coupled plasma optical emission spectrometry (ICP-OES) using an Agilent 5900 ICP-OES instrument (Santa Clara, CA, USA), while chloride and fluoride ions were quantified by ion chromatography (IC).

To complement the leaching assessment, the solid materials were characterized by determining moisture content, loss on ignition (LOI), total organic carbon (TOC), phenol index, BTEX compounds, polycyclic aromatic hydrocarbons (PAHs), polychlorinated biphenyls (PCBs), and mineral oil hydrocarbons (C10–C40). These analyses provided a comprehensive evaluation of the inorganic and organic constituents potentially affecting the environmental performance of the investigated composites.

The obtained results were compared with the limit values specified in the Regulation of the Minister of Economy of 16 July 2015 on waste acceptance criteria for landfilling. These limits were adopted solely as a conservative reference framework for assessing the potential mobility of contaminants and should not be interpreted as a direct assessment of the suitability or safety of the investigated materials for indoor construction applications. Since the intended application of the developed composites is in monolithic 3D-printed concrete elements, the leaching test represents a worst-case exposure scenario, as it is performed on crushed material possessing a substantially greater specific surface area than the final structural elements. Consequently, contaminant release measured under these laboratory conditions can be regarded as an upper-bound estimate of the environmental impact expected during practical service conditions.

#### 2.2.3. Assessment of Biological Durability Through Growth of Material-Associated Microorganisms

The objective of the experiment was to assess the specimens that had previously been exposed to outdoor environmental conditions for six months and could therefore contain viable microorganisms associated with their surface and pore structure. The microbial growth emerging from the specimens was evaluated using carbohydrate-based media to promote the growth and visualization of the pre-existing material-associated microorganisms. Two medium formulations were prepared by dissolving sucrose in distilled water at concentrations of 3% (*w*/*v*) and 6% (*w*/*v*) under continuous stirring. The resulting solutions were sterilized by autoclaving at 121 °C and 103 kPa (15 psi) for 20 min. Solidification of the media was achieved using a 4% (*w*/*v*) solution of Żelfix (Dr. Oetker, Gdańsk, Poland), a commercial pectin-based gelling agent containing sucrose, citrus pectin, citric acid, and sorbic acid. The Żelfix solution was prepared separately by boiling for 5 min and was aseptically added to the sterilized sucrose media after cooling. Following thorough mixing, the media were allowed to cool to approximately 45–50 °C and subsequently poured into sterile 120 mm polystyrene Petri dishes under a laminar-flow biosafety cabinet. The plates were left undisturbed under sterile conditions until complete gelation and solidification of the media.

**Figure 1:**
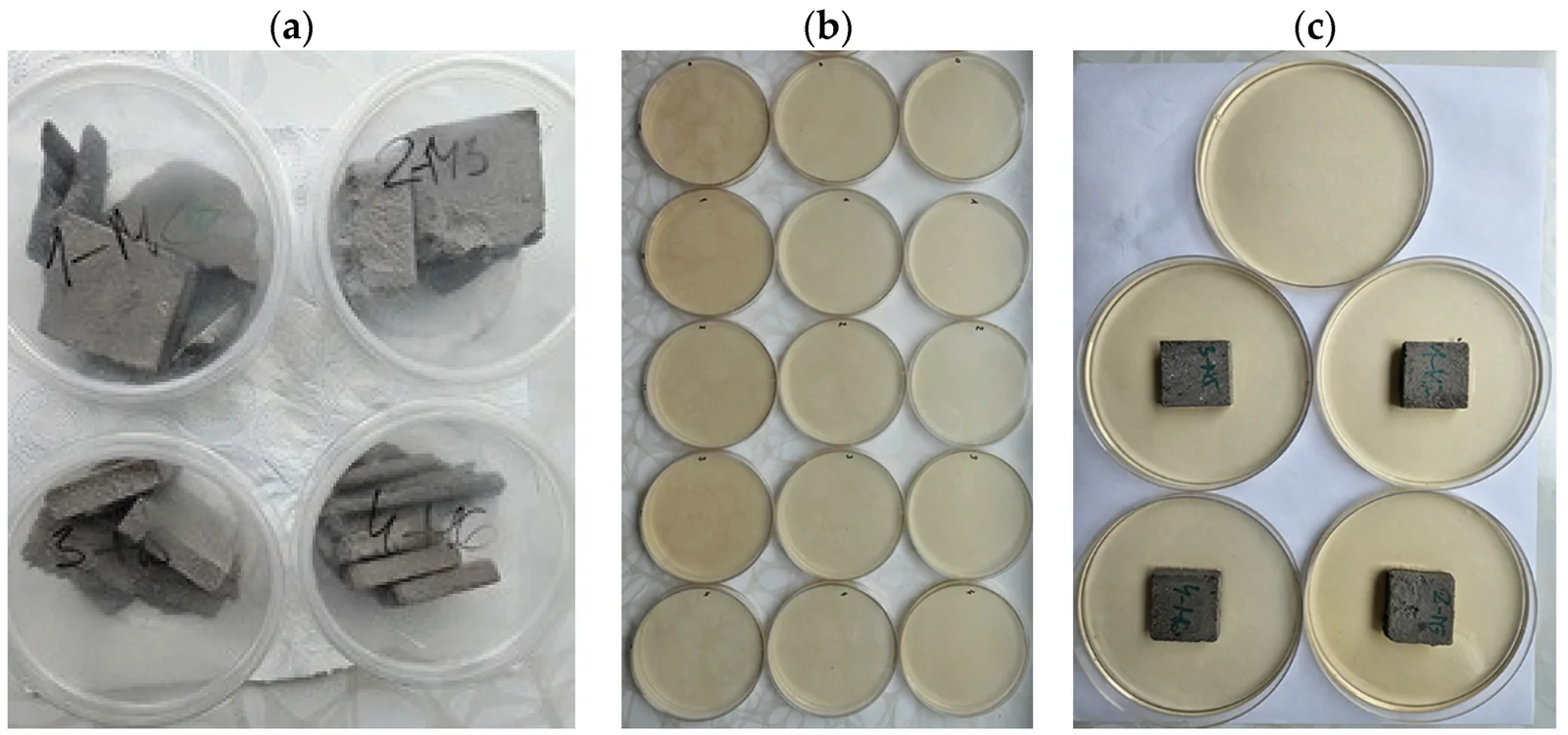
Experimental setup used for evaluating the growth of material-associated microorganisms on the surface of investigated composites. (**a**) Test specimens before exposure, (**b**) Petri dishes containing the culture media before the experiment, and (**c**) specimens placed on the culture medium at the start of the experiment. All samples were exposed to identical environmental and nutritional conditions throughout the test period, allowing direct comparison of material-dependent microbial development.

Rectangular specimens (50 × 50 mm) of the produced materials were aseptically placed on the surface of the solidified media. All specimens were 3D-printed and subsequently cut to obtain uniform, flat surfaces, ensuring comparable surface geometry and conditions for the assessment of potential microorganism growth. A positive control was not included in the experimental design, as the primary objective of the study was to compare the relative susceptibility of different materials to fungal growth under identical test conditions. Two levels of negative controls were employed: (i) sterile culture medium without samples was used to verify the absence of external contamination and (ii) all media contained a fungal growth-inhibiting substance (sorbic acid) to prevent unintended microbial contamination and to confirm the effectiveness of growth suppression. Together, the control ensured the validity of the experimental procedure and the reliability of the observed differences among the tested materials. For each material type, four independent replicates were prepared (Figure 1).

Following sample placement, the Petri dishes were sealed with laboratory parafilm and transferred to controlled environmental conditions. Growth of material-associated microorganisms was conducted for 28 days under ambient laboratory conditions at 23 ± 1 °C and relative humidity of 50 ± 10%.

The samples were visually inspected throughout the incubation period. Macroscopic changes, including the appearance of bacterial colonies, fungal mycelia, sporulation, pigmentation, and biofilm formation, were documented photographically using a Samsung Galaxy Z Fold5 smartphone camera (Suwon, Republic of Korea) under standardized lighting conditions.

Microbial growth emerging from the specimens on the media surface and/or material samples was evaluated qualitatively. Microorganisms were classified based on colony morphology into two principal groups: bacteria and fungi (yeasts and filamentous fungi).

To complement the visual assessment, a quantitative image analysis was performed using ImageJ software (version 1.54, National Institutes of Health, Bethesda, MD, USA). For each image, a region of interest (ROI) corresponding to the entire exposed specimen surface was manually defined to exclude the surrounding culture medium from the analysis. Images were preprocessed by color normalization and conversion to 8-bit grayscale suitable for segmentation. Microbial growth was identified using a color-based segmentation procedure, in which pixels corresponding to visible fungal growth and growth-related discoloration were separated from the unaffected material surface through thresholding. The segmented area was quantified and expressed as the percentage of the total ROI area covered by microbial growth. Four independent replicates were analyzed for each material, and the results were presented as the observed minimum-to-maximum percentage range to reflect inter-replicate variability.

## 3. Results and Discussion

### 3.1. Thermal Behavior of the Composite

**Figure 2:**
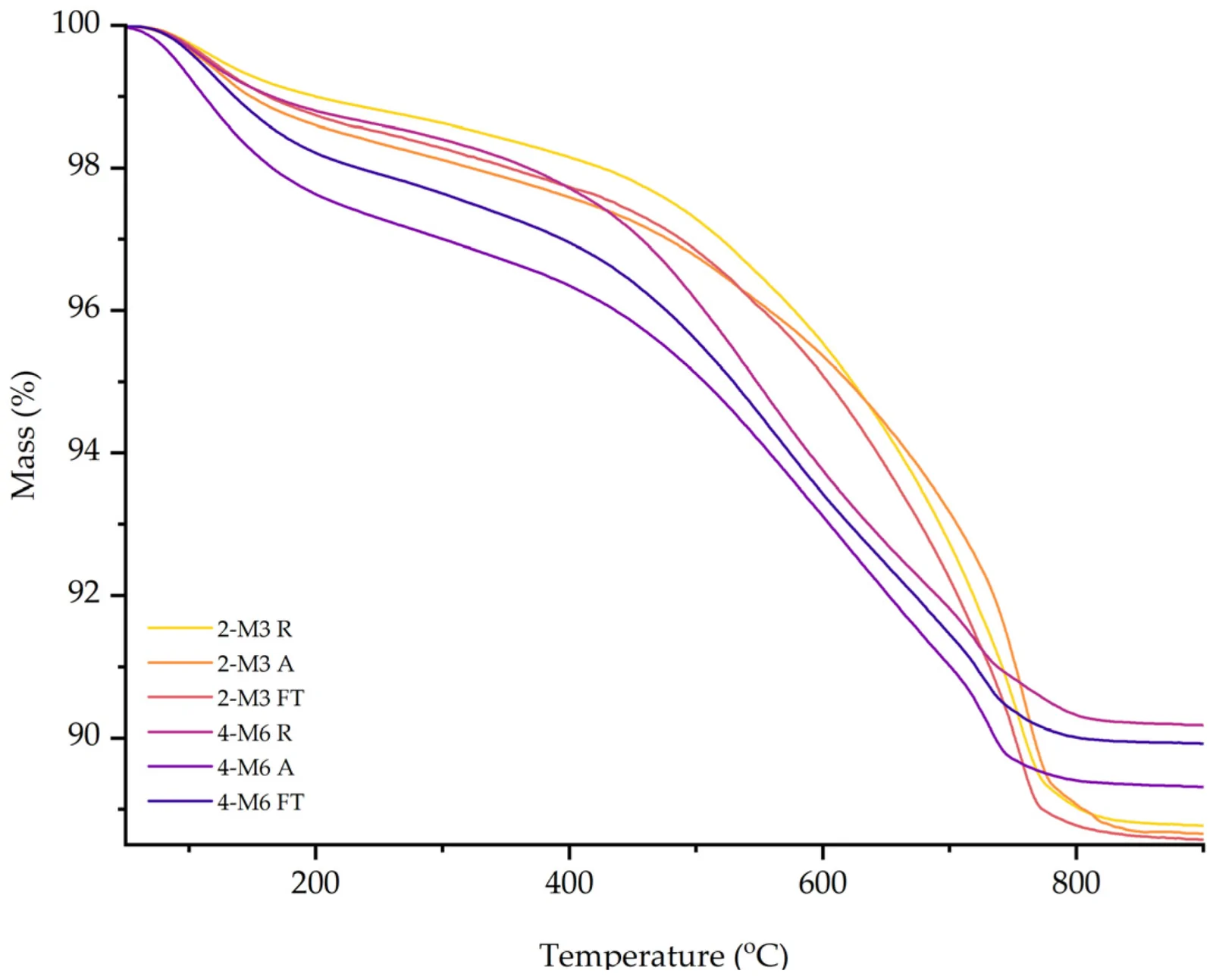
Representative thermogravimetric (TG) curves showing the thermal decomposition profiles of the fiber-reinforced foamed composites under different conditioning regimes (R—reference, A—atmospheric-aged, and FT—freeze–thaw).

**Figure 3:**
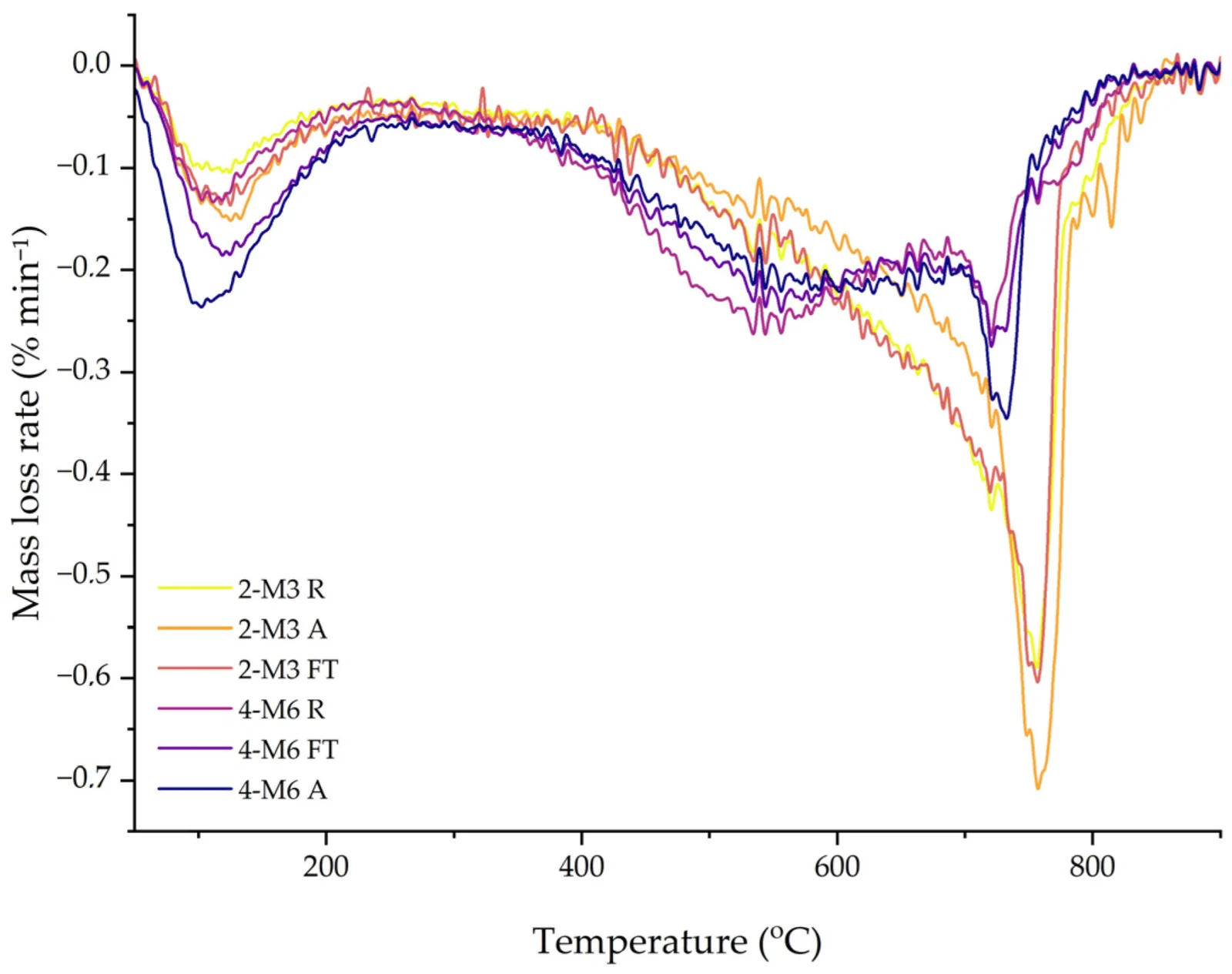
Representative derivative thermogravimetric (DTG) curves showing the characteristic decomposition peaks of the fiber-reinforced foamed composites under different conditioning regimes (R—reference, A—atmospheric-aged, and FT—freeze–thaw).

**Figure 4:**
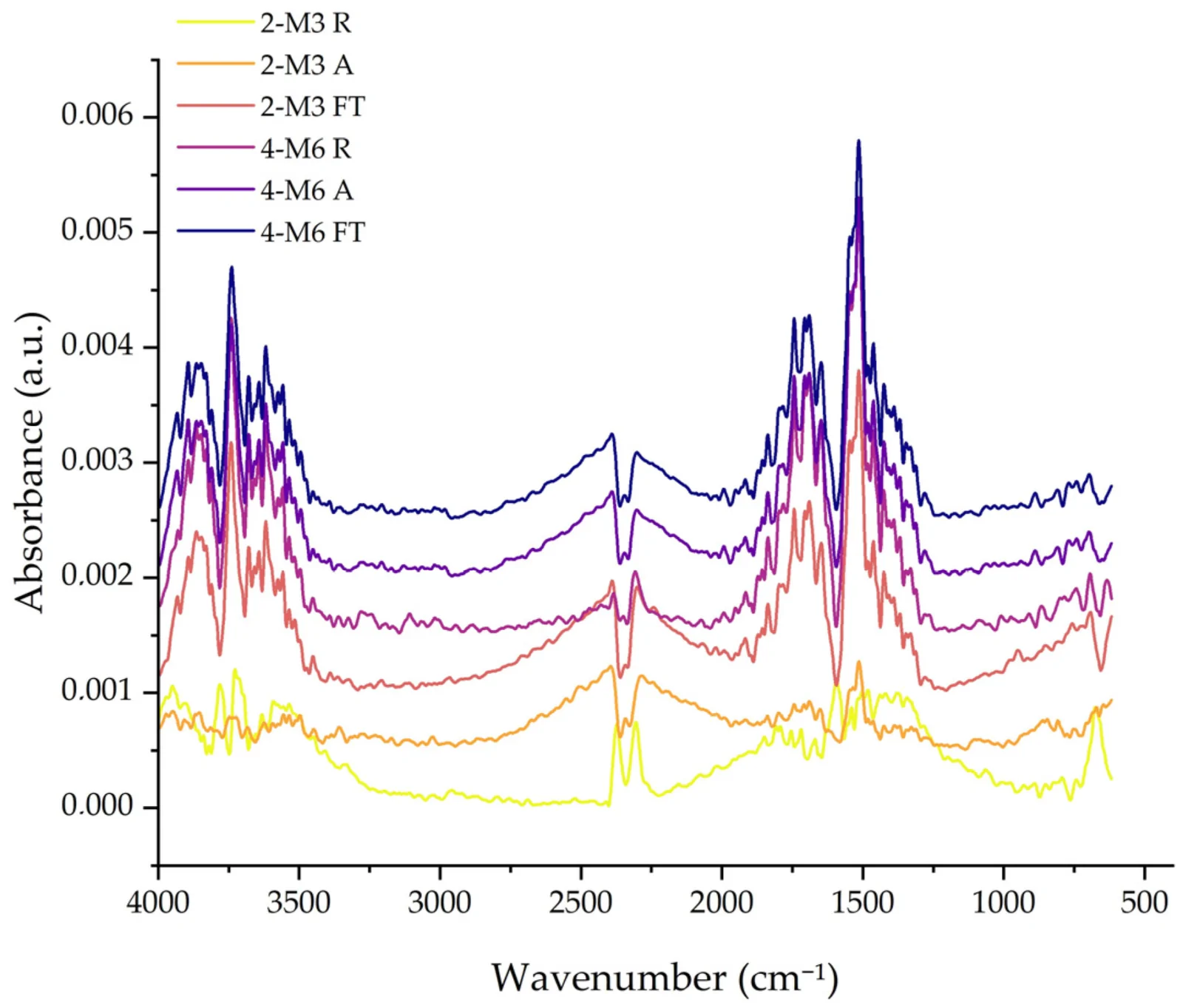
FTIR spectra of the gaseous products evolved during Stage I (100–126 °C) of thermal decomposition of the fiber-reinforced foamed composites under different conditioning regimes (R—reference, A—atmospheric-aged, and FT—freeze–thaw).

**Figure 5:**
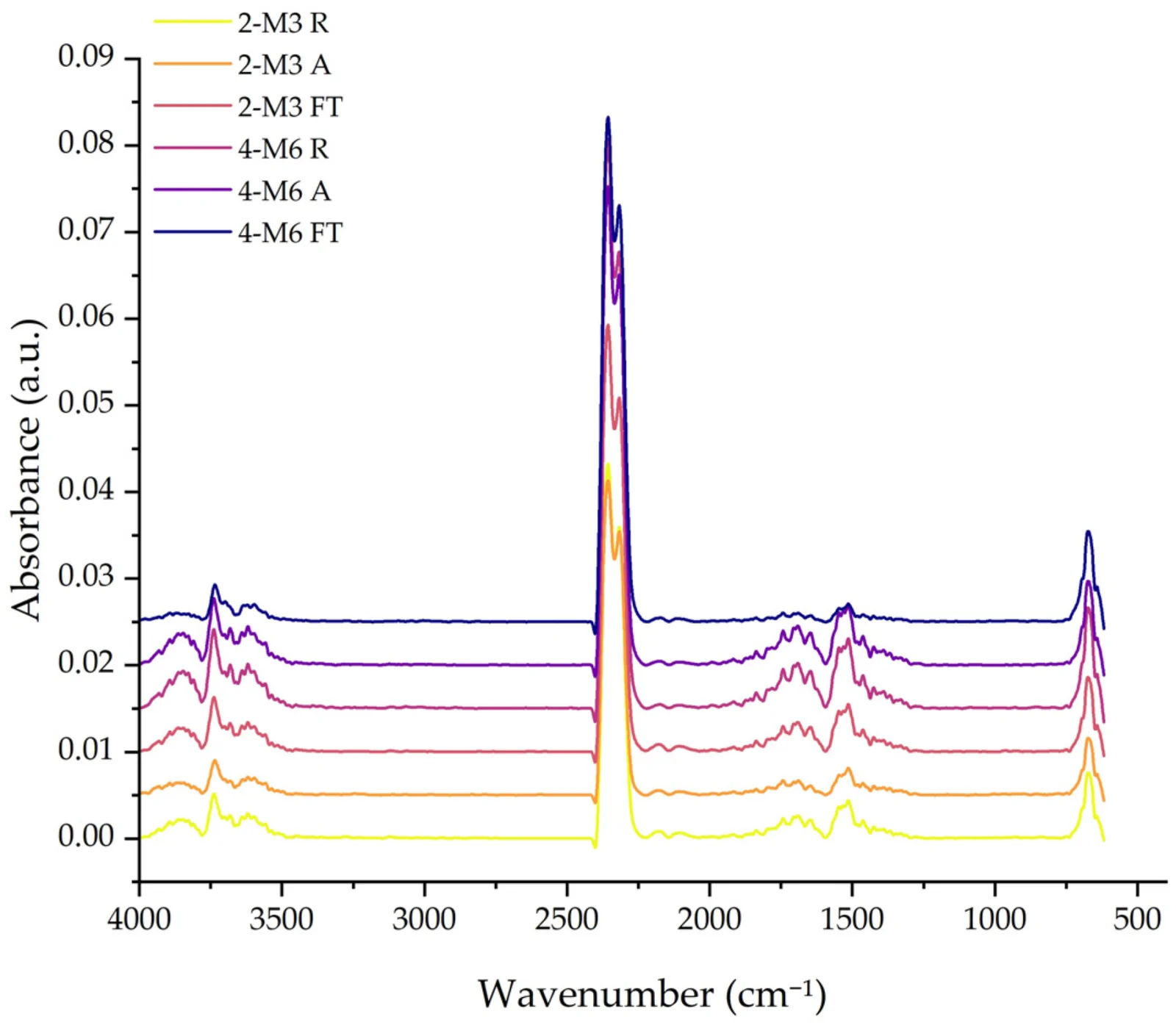
FTIR spectra of the gaseous products evolved during Stage II (534–556 °C) of thermal decomposition of the fiber-reinforced foamed composites under different conditioning regimes (R—reference, A—atmospheric-aged, and FT—freeze–thaw).

**Figure 6:**
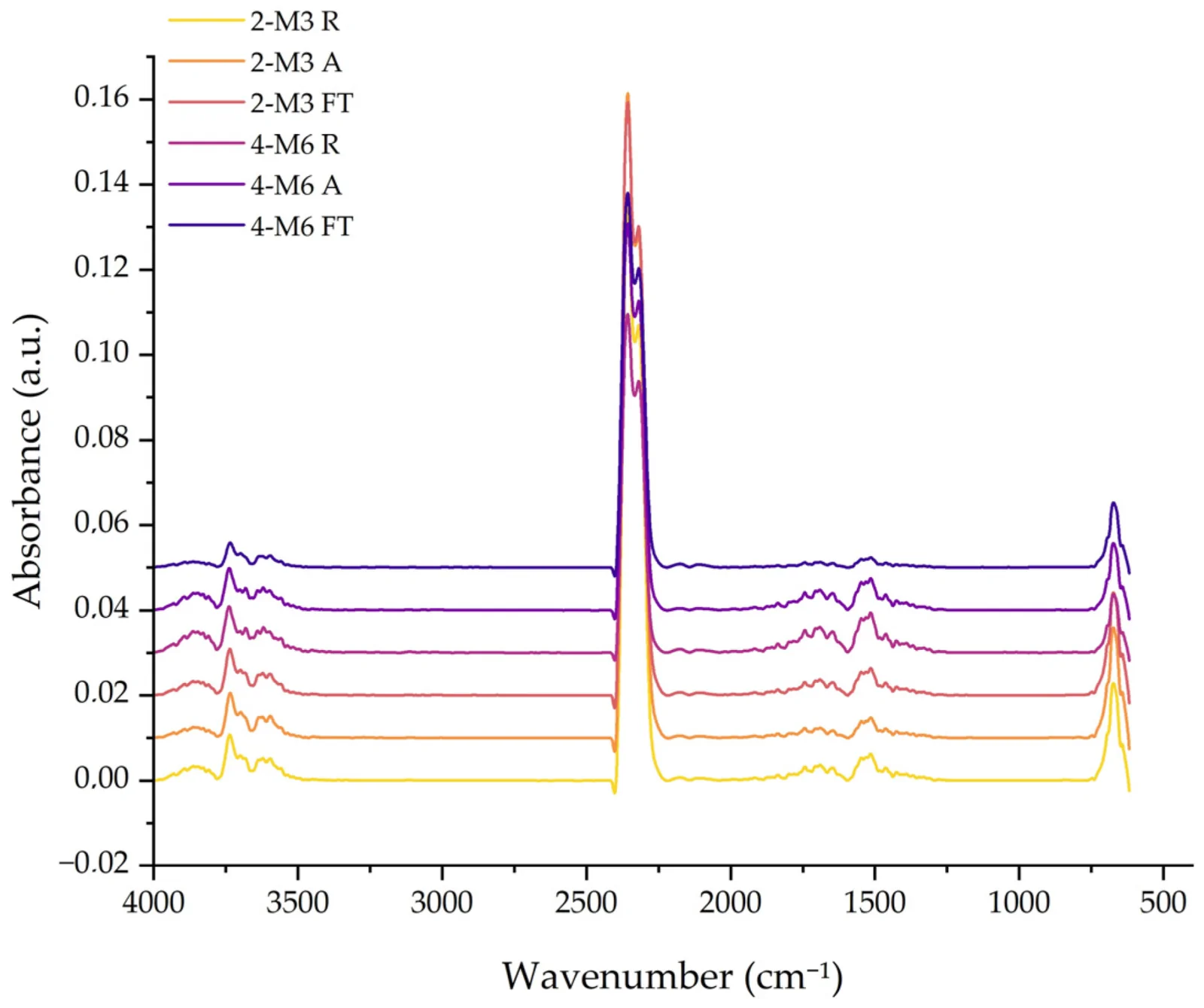
FTIR spectra of the gaseous products evolved during Stage III (720–757 °C) of thermal decomposition of the fiber-reinforced foamed composites under different conditioning regimes (R—reference, A—atmospheric-aged, and FT—freeze–thaw).

The thermal decomposition behavior of the investigated 3D-printed fiber-reinforced foamed cement composites was examined using simultaneous thermogravimetric analysis (TG), derivative thermogravimetry (DTG), and coupled Fourier-Transform Infrared Spectroscopy (TG-FTIR). The TG and DTG curves are presented in Figure 2 and Figure 3, whereas the FTIR spectra recorded at characteristic decomposition temperatures are shown in Figure 4, Figure 5 and Figure 6.

The TG curves demonstrated that all specimens, reference (R), atmospheric-aged (A), and freeze–thaw conditioned (FT), followed the typical three-stage thermal decomposition pathway characteristic of hydrated cementitious systems. No additional decomposition steps or unexpected thermal events were detected, indicating that neither six-month natural atmospheric exposure nor freeze–thaw cycling altered the fundamental degradation mechanism. Although the overall decomposition sequence remained consistent, distinct differences in the intensity of individual thermal events were observed between the cement-based composite (2-M3) and the alkali-activated composite (4-M6). The 2-M3 specimens exhibited highly similar TG profiles across all conditioning regimes, whereas the 4-M6 specimens showed more pronounced variations in mass loss, particularly within the temperature range associated with the decomposition of hydration products and carbonate phases. These findings suggest that the binder chemistry had a greater influence on thermal behavior than environmental conditioning.

The characteristic thermal decomposition parameters obtained from TG-DTG analysis were summarized in Table 6. All composites exhibited the same three-stage degradation mechanism; however, noticeable differences were observed in the characteristic temperatures and corresponding mass losses, further confirming the dominant role of binder composition. The first decomposition stage, mainly associated with the release of physically adsorbed and weakly bound water, may also include partial dehydration of hydration products. DTG peak temperatures varied only slightly among the specimens, while mass loss ranged from 1.25 to 2.81%. The highest value was recorded for the atmospheric-aged 4-M6 specimen (A), whereas all remaining samples exhibited mass losses below ~2.2%. The narrow range of values suggests that environmental exposure had only a minor effect on the amount of physically retained moisture.

More substantial differences were observed during the second decomposition stage, mainly associated with dehydration and dehydroxylation of hydration products. The characteristic DTG peak temperatures ranged from approximately 534 to 556 °C. For the 2-M3 composites, mass loss remained nearly constant (~2.5%), and the atmospheric-aged specimen (A) did not exhibit a clearly distinguishable DTG peak, indicating overlap between the decomposition of hydration products and adjacent thermal events. In contrast, the 4-M6 composites showed significantly greater variability. The reference (R) and freeze–thaw conditioned (FT) specimens exhibited markedly higher mass losses (6.48% and 5.53%, respectively), whereas the atmospheric-aged specimen (A) showed only 2.80%. This may suggest that prolonged atmospheric exposure affected the amount or thermal stability of hydrated phases in the alkali-activated binder. It should be noted that partial overlap with the thermal decomposition of merino wool fibers and organic constituents of the foaming agent cannot be excluded within this temperature range.

The most pronounced differences between the binder systems were observed during the third decomposition stage associated with carbonate decomposition. The 2-M3 specimens decomposed at approximately 756–757 °C and exhibited substantially higher mass losses (7.49–9.62%), whereas the 4-M6 specimens decomposed at lower temperatures (720–733 °C) and showed significantly lower mass losses (1.89–3.10%). These results suggest differences in both the quantity and thermal stability of carbonate phases formed in the two binder systems. Environmental conditioning had only a minor influence within each mixture series compared with the dominant effect of binder composition.

Despite these differences, all composites exhibited high thermal stability, as confirmed by the residual mass at ~900 °C, which ranged from 88.56 to 90.17%. Consequently, the total mass loss did not exceed ~10–11%, demonstrating that both lightweight foamed composites retained the majority of their inorganic mineral skeleton after thermal decomposition.

The characteristic temperatures identified from the DTG curves corresponded closely to the maxima observed in the Gram–Schmidt profiles, confirming that the highest rate of gaseous evolution coincided with the principal decomposition reactions. These temperatures were subsequently those of the evolved gaseous species released during thermal decomposition rather than directly characterizing the structure of the solid binder.

The FTIR spectra recorded at the characteristic temperature of the first decomposition stage (Figure 4) were dominated by absorption bands assigned to water vapor. The broad absorption observed within the 3700–3500 cm^−1^ region corresponds to O–H stretching vibrations, while the weaker band near 1600 cm^−1^ is attributed to H–O–H bending vibrations [56,57]. Despite the similar spectral pattern observed for all specimens, noticeable differences in band intensity were identified. The atmospheric-aged 2-M3 specimen (2-M3 A) exhibited the highest absorbance of water-related bands, whereas the spectra of the alkali-activated 4-M6 specimens remained relatively similar irrespective of the conditioning regime. These observations suggest that environmental exposure influenced the amount of physically retained moisture more strongly in the Portland cement composite than in the alkali-activated system, while the mechanism of water release remained unchanged, consistent with the thermal dehydration behavior commonly reported for hydrated cementitious materials [58].

The second decomposition stage, centered between approximately 540 and 560 °C, is mainly attributed to the dehydroxylation of hydrated cement phases, primarily calcium hydroxide, together with the progressive dehydration of C–S–H and C–A–S–H gels [59]. However, partial overlap with the thermal decomposition of merino wool fibers and the organic constituents of the foaming agent cannot be excluded. According to previous thermogravimetric studies of hydrated cementitious materials, the thermal interval between approximately 400 and 550 °C is predominantly associated with portlandite dehydroxylation, while partial overlap with the dehydration of C–S–H and C–A–S–H phases may occur depending on the binder composition and experimental conditions [60]. Therefore, the thermal event observed in the present study was interpreted as the combined effect of these overlapping decomposition processes rather than being assigned exclusively to a single hydration product. The TG–DTG analysis suggests that this stage was more pronounced in the alkali-activated composites, indicating differences in the quantity and stability of the hydration products formed within the two binder systems [61]. The interpretation is consistent with previous TG/HT-XRD studies showing that overlapping decomposition of the hydrated cement phases may occur within this temperature interval [62].

The corresponding FTIR spectra collected at this temperature (Figure 5) show a substantial decrease in the intensity of water-related absorption bands compared with the first stage, confirming that most physically adsorbed water had already been removed. At the same time, weak residual absorption associated with hydroxyl-containing species remained detectable, reflecting the release of chemically bound water during decomposition of hydration products. Compared with the reference specimens, only moderate differences were observed after atmospheric exposure and freeze–thaw cycling, suggesting that environmental conditioning had only a limited influence on the nature of the hydrated phases [63].

The third decomposition stage, occurring between approximately 720 and 760 °C, represented the dominant thermal event for all investigated specimens and corresponds to the decarbonation of carbonate phases formed during hydration and subsequent carbonation [64,65]. The FTIR spectra recorded at this temperature (Figure 6) were dominated by the characteristic carbon dioxide absorption bands located near 2350 cm^−1^ (asymmetric stretching vibration) and approximately 670 cm^−1^ (bending vibration), confirming that CO_2_ evolution constituted the principal gaseous product released during this stage [66]. A comparison of the spectra revealed noticeable differences in CO_2_ evolution between the investigated composites. The 2-M3 specimens, particularly the atmospheric-aged specimen (2-M3 A), exhibited the highest intensity of the CO_2_ absorption bands, indicating a larger amount of thermally decomposable carbonate phases. In contrast, the M6 specimens showed considerably lower CO_2_ absorbance, although the spectral profiles remained highly similar for the reference, atmospheric-aged, and freeze–thaw conditioned specimens. This behavior is consistent with the TG–DTG results, which demonstrated that the binder type exerted a greater influence on carbonate decomposition than the applied environmental exposure.

Overall, the combined TG–DTG–FTIR analysis indicates that both composite systems followed the characteristic thermal decomposition pathway of hydrated cementitious materials, consisting of three main thermal decomposition stages associated with moisture release, dehydroxylation of hydrated phases, and carbonate decomposition. It should be emphasized that the FTIR spectra presented in Figure 4, Figure 5 and Figure 6 correspond to the gaseous products evolved during thermal decomposition rather than to the solid binder itself. Therefore, the spectra provide indirect information on the decomposition reactions occurring during heating and should not be interpreted as direct evidence of the structure, stability, local bonding environment, or degree of hydration of the solid cementitious matrix. The observed differences are primarily related to the intensity of individual thermal events rather than to changes in the overall decomposition mechanism itself. Environmental conditioning produced only limited changes in the evolved gaseous products, whereas the type of binder appeared to have a greater influence on the thermal behavior and gas evolution characteristics of the investigated composites.

### 3.2. Environmental Safety

The environmental performance of the developed materials was assessed through leaching tests and chemical characterization to evaluate their potential impact during service life and after the end of their life cycle. Considering the intended application of the investigated composites as materials for 3D-printed concrete buildings, particular attention was paid to the mobility of inorganic contaminants and the presence of hazardous organic compounds. The leachates obtained from both the non-activated and activated materials exhibited a strongly alkaline character, with pH values of 11.0 and 11.4, respectively (Table 7). Such values are typical of cement-based and alkali-activated materials due to the presence of calcium hydroxide and alkaline reaction products [67]. Although high alkalinity generally promotes the immobilization of many heavy metals through precipitation and adsorption processes, it may simultaneously increase the solubility of amphoteric elements, particularly chromium [68].

The analyses of the solid materials revealed very low concentrations of organic contaminants. The BTEX content amounted to 41 μg/kg for the non-activated material and 35 μg/kg after activation, while polycyclic aromatic hydrocarbons (PAHs), polychlorinated biphenyls (PCBs), and mineral oil hydrocarbons were detected only at trace levels. The total organic carbon (TOC) did not exceed 0.60 wt.% for both materials. These results indicate that the investigated composites do not constitute a significant source of organic pollutants, which is particularly important for construction materials intended for residential applications.

Under highly alkaline conditions, most divalent metal cations are effectively immobilized through precipitation as hydroxides or incorporation into calcium silicate hydrate (C-S-H) or aluminosilicate gels [69]. Consequently, the leaching of cadmium, lead, nickel, mercury, zinc, barium, and selenium remained very low or below the analytical detection limits. Results presented in Table 8 confirm that both binder systems provide an effective chemical barrier limiting the release of potentially hazardous trace metals.

The highest mobility among the investigated elements was observed for chromium. Chromium concentrations reached 3.6 mg/kg for the non-activated composite and 4.0 mg/kg after alkali activation. This behavior is consistent with the amphoteric nature of chromium species. Under strongly alkaline conditions, chromium is predominantly present as soluble chromate oxyanions (CrO_4_^2−^), which exhibit lower affinity for cement hydration products than cationic metals. Therefore, even though the material matrix remains chemically stable, elevated pH may promote chromium mobility to a greater extent than for other heavy metals. The relatively small increase after alkali activation suggests that the higher alkalinity rather than deterioration of the matrix governs chromium release [70,71]. A similar tendency was observed for arsenic and antimony, whose leaching increased after alkali activation. Unlike most metallic cations, arsenic and antimony occur mainly as oxyanionic species under alkaline conditions. Their adsorption on hydrated cement phases is considerably weaker than that of cationic metals, resulting in increased mobility despite the highly alkaline environment [72,73]. The measured concentrations remained relatively low, indicating that although alkali activation influences the release of these elements, the newly formed aluminosilicate matrix still provides effective immobilization. Copper exhibited a noticeable increase in leaching from negligible concentrations in the non-activated composite to 4.5 mg/kg after activation. Although copper generally demonstrates limited mobility in Portland cement systems, the alkali activation process modifies the pore solution chemistry and promotes dissolution of precursor phases during the early stages of geopolymerization [74]. Part of the released copper may therefore remain temporarily in the pore solution before becoming incorporated into the developing aluminosilicate gel network. This mechanism has been reported for several alkali-activated binders produced from industrial by-products and does not necessarily indicate poor long-term environmental stability. Among the anions, sulfate exhibited the highest release, increasing from 2700 to 4700 mg/kg following alkali activation [75,76]. The increased sulfate release is likely associated with the dissolution of readily soluble sulfate-bearing phases together with the high ionic strength of the pore solution characteristic of alkali-activated binders, rather than indicating degradation of the hardened matrix itself. Simultaneously, total dissolved solids (TDS) increased markedly to approximately 18,000 mg/kg, reflecting the greater concentration of soluble alkali ions introduced during activation. Importantly, the increase in TDS should not be interpreted as evidence of enhanced release of hazardous contaminants but rather as a consequence of the elevated alkali content characteristic of alkali-activated systems [77,78].

Comparison with the WAC limit values demonstrated that the majority of the investigated parameters remained well below the regulatory thresholds adopted as a conservative reference framework. Only chromium and sulfate exhibited comparatively higher mobility, although their release remained consistent with the behavior commonly reported for highly alkaline cementitious and alkali-activated materials [79]. Overall, the results indicate that the incorporation of recycled Merino wool fibers did not significantly influence the leaching behavior of the developed composites. As commonly reported for cementitious and alkali-activated materials, contaminant mobility was governed predominantly by binder chemistry, pore solution composition, and the resulting physicochemical characteristics of the matrix.

The observed increase in the leaching of selected elements after activation is most likely associated with changes in the material microstructure during the alkali activation process. The dissolution of amorphous aluminosilicate phases and the formation of new reaction products may temporarily enhance the availability of certain ions before they become fully incorporated into the hardened binder matrix [77,80]. Similar behavior has been reported for other alkali-activated materials containing industrial by-products, where increased early-stage leaching does not necessarily correspond to reduced long-term environmental stability [81].

It should be emphasized that the present study employed the standardized 24 h batch leaching test (PN EN 12457-3), which is intended as a comparative environmental screening method and represents a conservative assessment of contaminant mobility. The test is performed on crushed material with a substantially greater specific surface area than monolithic elements produced by 3D concrete printing. Consequently, under actual service conditions, contaminant transport is expected to be considerably lower owing to the dense cementitious matrix, the limited contact surface exposed to the environment, and the continuing hydration and maturation processes that further reduce pore connectivity. Therefore, the obtained results should be interpreted as a worst-case scenario rather than as a direct prediction of contaminant release from full-scale printed structural elements [82,83]. It should also be noted that the present study was limited to a standardized 24 h batch leaching test and therefore does not provide information on the long-term kinetics of contaminant release. Comprehensive long-term environmental investigations, including several years of natural outdoor exposure of the developed composites, are currently underway and will provide further insight into the long-term environmental performance of these materials under realistic service conditions.

From the perspective of the standardized environmental assessment performed in this study, both investigated materials exhibited low concentrations of hazardous organic compounds and limited mobility of most analyzed elements. The highest leaching values were observed for chromium and sulfates, while the alkali-activated composite additionally showed increased leaching of arsenic, antimony, and copper. These findings suggest that further optimization of the binder composition, particularly the type and dosage of the alkaline activator, may further improve the immobilization efficiency of these elements. Considering that the leaching assessment was performed using a conservative worst-case scenario based on crushed specimens, the developed composites exhibited promising environmental performance under the investigated laboratory conditions. However, their long-term environmental behavior under realistic service conditions requires further verification through ongoing long-term exposure studies. The increased leaching of selected elements observed in the alkali-activated composites indicates that further optimization of the binder system may improve their environmental performance. According to previous studies, the mobility of potentially hazardous elements can be reduced by optimizing the type and dosage of the alkaline activator, adjusting the silicate modulus, incorporating supplementary cementitious materials (e.g., fly ash), or introducing immobilization additives capable of enhancing heavy-metal binding within the aluminosilicate matrix [84,85]. Previous studies have shown that optimizing the sodium silicate modulus and incorporating suitable additional precursor materials can significantly improve the immobilization efficiency of alkali-activated binders by reducing the mobility of selected potentially hazardous elements [86]. The verification of these strategies was beyond the scope of the present study but constitutes the next stage of the ongoing research.

### 3.3. Biological Durability

**Figure 7:**
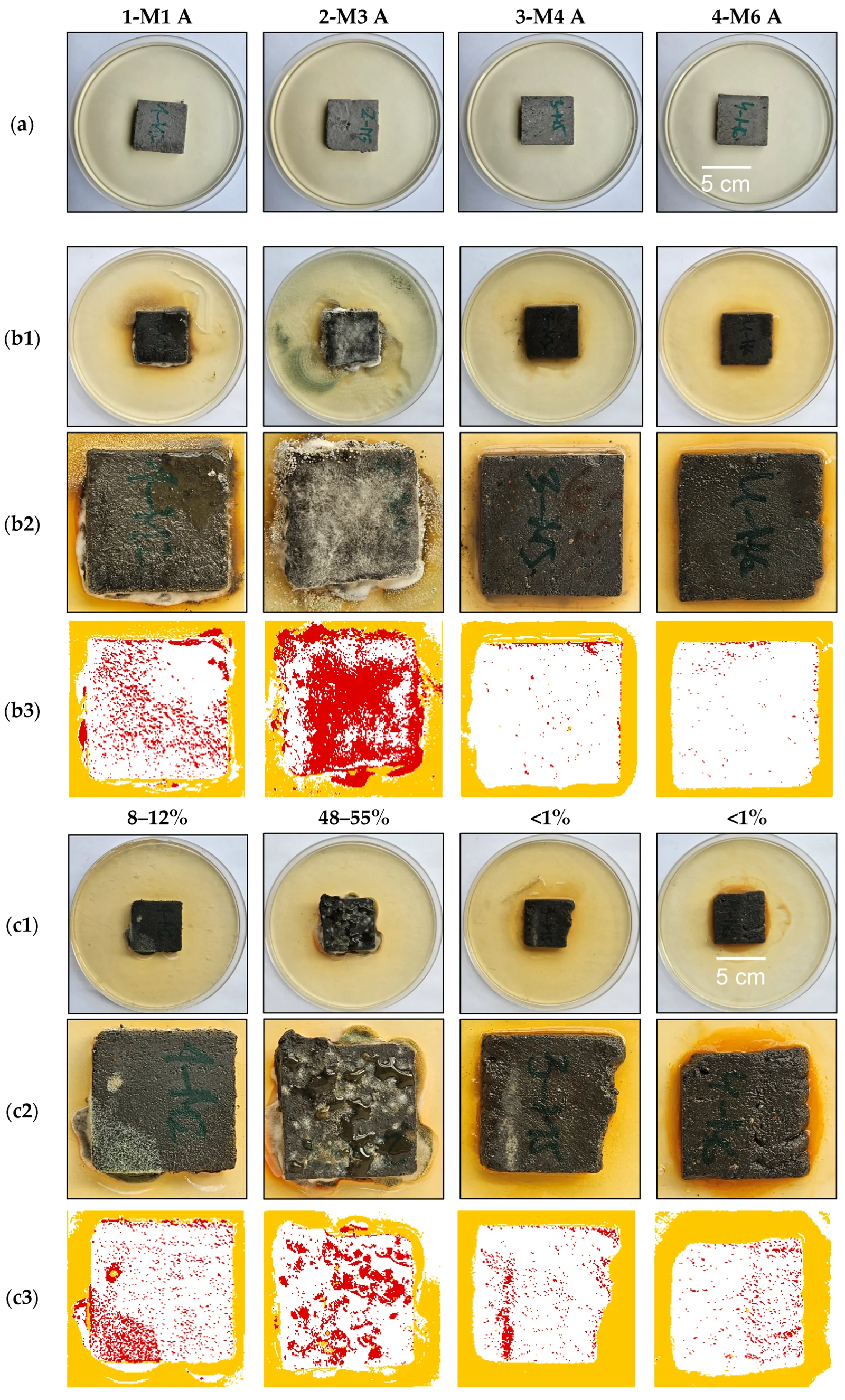

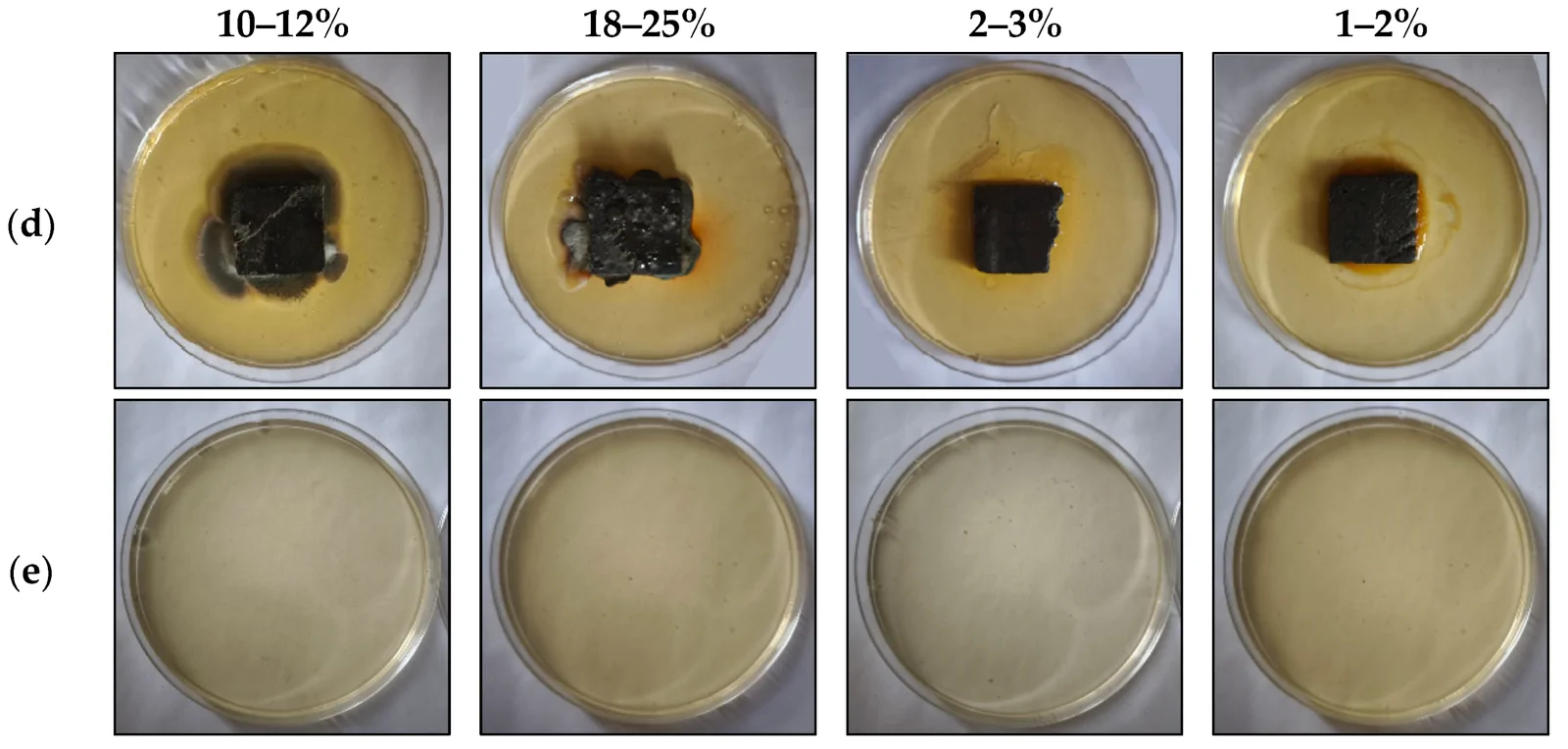
Representative photographs of the printed investigated fiber-reinforced foamed composites in non-activated and activated conditions during the assessment of growth of material-associated microorganisms: (**a**) samples placed on the culture medium at the beginning of the experiment, (**b1**–**b3**) samples after 28 days under fungal growth conditions on a medium supplemented with 3% sucrose, (**c1**–**c3**) samples after 28 days under fungal growth conditions on a medium supplemented with 6% sucrose, (**d**) samples after 56 days under fungal growth conditions on a medium supplemented with 6% sucrose, and (**e**) negative control after 56 days. (**b3**,**c3**) digital segmentation of biodeterioration patterns; red regions indicate areas classified as fungal growth emerging from the specimens based on image texture and color characteristics (percentage: (Area red/(Area red + A white)) × 100); white areas correspond to unaffected material surfaces and background; the segmentation was performed for comparative visualization of microbial development among the investigated material with ImageJ software (version 1.54). Four independent replicates were evaluated for each variant, and the photographs shown in the figure represent typical examples of the observed response, while the results of quantitative image-based assessment, performed to illustrate the observed patterns of microbial growth, are presented as percentage ranges reflecting the variability observed among the replicates.

The microbiological approach enabled assessment of both spontaneous microbial contamination associated with the specimens and the intrinsic resistance of the materials to microbial growth under conditions representative of environmental exposure. Figure 7 presents representative photographs of the tested materials after 28 days of incubation on sucrose-based semi-solid media. Prior to incubation (Figure 7a), all specimens exhibited a uniform appearance, with no visible signs of microbial contamination or surface deterioration.

Macroscopic observations revealed that fungal growth preferentially originated at the interface between the material and the nutrient medium, subsequently spreading across the specimen surface. The dominant manifestations of microbial growth included white and gray mycelial structures, occasional green-pigmented zones, and localized discoloration of the surrounding medium. No extensive bacterial biofilms or mucilaginous colonies were visually observed under the applied incubation conditions. The resistance of the analyzed composites to resident microbial communities was evaluated using a semi-quantitative visual rating scale (Table 9), ranging from 0 (no visible microbial growth) to 5 (very extensive microbial growth covering more than 90% of the sample surface). The assessment performed after 28 days of incubation (Table 10) revealed clear differences in the susceptibility of the investigated mixtures to microbial growth (Figure 7). Among the tested materials, sample 2-M3 A exhibited the highest degree of microbial growth emerging from the specimens, as indicated by visual changes in both the material surface and the surrounding medium. Dense white mycelium accompanied by green pigmentation was evident, indicating the development of filamentous fungal communities. The sample reached the maximum rating (5) for both media. Sample 1-M1 A showed only limited fungal development, predominantly confined to the specimen margins, and was therefore assigned growth of material-associated microorganism ratings of 2 and 1 on media 1 and 2, respectively. In contrast, no visible microbial growth was observed for the alkali-activated samples 3-M4 A and 4-M6 A (rating 0 for both media), while slight discoloration of the surrounding medium was detected. This indicated aseptic conditions in the material interior and surface under applied test conditions. A complementary ImageJ-based image analysis was performed to visualize and quantify microbial growth patterns (Figure 7(b3,c3)). While the percentage surface coverage values obtained by this method exhibited a narrower distribution, the relative ordering of the specimens and the trends observed across the investigated materials were in good agreement with those identified using the visual assessment scale. This consistency between independent evaluation methods strengthens confidence in the validity and reproducibility of the observed results.

Since 1-M1 A and 2-M3 A were prepared without alkaline activation, while 3-M4 A and 4-M6 A were alkali-activated, the obtained results suggest that alkaline activation played a role in preventing the growth of material-associated microorganisms. Similar antifungal behavior has been reported for alkali-activated slag systems [87]. The enhanced resistance of 3-M4 A and 4-M6 A may be associated with changes in the composition and microstructure of the binder, including the formation of a denser matrix with reduced moisture availability, both of which are generally considered unfavorable for the growth of resident microbial communities [88]. Additionally, 2-M3 A, which contained a higher proportion of moisture-retaining merino fibers, could promote local water accumulation and fungal attachment. Because the sucrose-based media provided an excess external carbon source, microbial growth emerging from the specimens was not nutrient-limited. Therefore, differences in fungal coverage reflected the physicochemical properties of materials. Particularly, the lower visible growth on alkali-activated specimens may be related to pH, pore structure, moisture availability, surface characteristics, fiber distribution, or several combined factors. Nevertheless, merino fibers in the 2-M3 A sample may have also acted as an additional organic substrate after prolonged exposure [89,90].

These findings demonstrate that alkali activation can substantially improve the durability of cementitious composites intended for use in environments where biological deterioration may occur.

It is worth mentioning that all tested materials were evaluated using the same culture medium formulation containing identical amounts of Żelfix and, consequently, the same concentration of sorbic acid. Visible microbial growth was observed on several specimens, indicating that the sorbic acid concentration present in the medium was insufficient to prevent fungal development under the applied test conditions. The fungal susceptibility to sorbic acid differs substantially among taxonomic groups and even among individual species and strains. Therefore, the differences in microbial development are more likely attributable to the intrinsic physicochemical properties of the tested materials, including surface characteristics, alkalinity, moisture retention capacity, and nutrient availability, which collectively influence the establishment, selection, and proliferation of specific microbial communities. These findings are important from the perspective of construction durability and stability. Fungal growth emerging from the specimens represents the first stage of biodeterioration and may lead to discoloration, moisture accumulation, and progressive degradation of material surfaces [91]. Yakovleva et al. [92] demonstrated active fungal deterioration of concrete materials, while Andersen et al. [93] showed that fungal communities readily develop on susceptible building materials under favorable moisture conditions.

## 4. Conclusions

1. The FTIR spectra of all investigated composites exhibited the same characteristic absorption bands, indicating that alkali activation produced similar reaction products regardless of the subsequent exposure conditions. Neither calcium hydroxide nor freeze–thaw cycling resulted in the appearance of new absorption bands or the disappearance of existing ones, suggesting that the fundamental chemical structure of the alkali-activated binder remained stable.
2. Both binder systems followed the characteristic three-stage thermal decomposition pathway of hydrated cementitious materials, with residual masses exceeding 88.5 wt.% after heating to approximately 900 °C, indicating high thermal stability irrespective of the applied environmental conditioning.
3. Only minor variations in the intensity of selected absorption bands were observed after environmental exposure, indicating limited changes in the degree of hydration and the local bonding environment rather than significant degradation of the aluminosilicate network. These results confirm the good chemical stability of the developed alkali-activated composites under the investigated service conditions.
4. The investigated recycled fiber-reinforced foamed composites exhibited favorable environmental performance, characterized by TOC contents not exceeding 0.60 wt.%**,** low concentrations of hazardous organic compounds (BTEX: 35–41 μg/kg, PAHs < 0.85 mg/kg, PCBs < 0.007 mg/kg), and limited leaching of most analyzed elements under the investigated laboratory conditions.
5. Alkali activation increased the leachability of selected constituents, particularly chromium (3.6 → 4.0 mg/kg), arsenic (<0.02 → 1.2 mg/kg), antimony (0.073 → 0.26 mg/kg), copper (0.85 → 4.5 mg/kg), sulfates (2700 → 4700 mg/kg), and total dissolved solids (3700 → 18,000 mg/kg).
6. The high alkalinity of both binder systems effectively immobilized the majority of heavy metals, whereas the comparatively higher mobility of chromium and sulfate was consistent with the behavior commonly reported for highly alkaline cementitious and alkali-activated materials. Despite these differences, the leaching values of the majority of analyzed trace elements remained well below the regulatory limits specified in the Regulation of the Minister of Economy of 16 July 2015. The standardized batch leaching test performed on crushed specimens represents a conservative worst-case assessment based on waste acceptance criteria. Consequently, the obtained results should be interpreted as an environmental screening of contaminant mobility under standardized laboratory conditions rather than as a direct prediction of contaminant release from monolithic 3D-printed structural elements. Owing to their lower exposed surface area and more compact structure, contaminant release from monolithic elements is expected to be lower under actual service conditions; however, this assumption requires verification through dedicated long-term environmental exposure and leaching studies. Therefore, the present results indicate a low leaching potential under the investigated laboratory conditions but should not be interpreted as a comprehensive assessment of the suitability of the investigated composites for indoor applications. Such an evaluation would require additional long-term durability and indoor environmental performance studies. The results indicate that the binder composition, particularly the alkaline activator dosage, should be regarded as the key parameter controlling contaminant immobilization during mixture design and should therefore be carefully optimized.
7. The preliminary microbiological assessment suggested higher resistance of the alkali-activated composites to the growth of material-associated microorganisms, whereas more extensive development of resident microbial communities was observed in specimens providing favorable moisture conditions and biodegradable constituents. Preliminary observations regarding the biological durability of the material indicated that the development of resident microbial communities may be related to alkalinity, porosity, moisture, surface characteristics, chemical composition, or several combined factors.

## Figures and Tables

**Table 1:** Expected environmental benefits associated with the recycled constituents used in the investigated composites.

Recycled Constituent	Source	Replaced Material	Expected Sustainability Benefit	Reference
Fly ash	Coal combustion by-product	Portland cement	Lower clinker consumption, reduced embodied CO_2_ emissions, industrial by-product utilization	[20]
Coal slag	Metallurgical by-product	Mineral filler/aggregate	Valorization of industrial waste and reduced landfill disposal	[21]
Ground brick waste	Construction and demolition waste	Natural aggregate	Reduced extraction of virgin aggregates and construction waste recycling	[22]
Recycled merino wool fibers	Textile industry waste	Virgin reinforcing fibers	Textile waste valorization and substantially lower carbon footprint than virgin wool fibers	[23]

**Table 2:** Constituents of the investigated 3D-printable fiber-reinforced foamed concrete and their functions.

Constituent	Material	Function in the Composite	Sustainability Contribution
Binder	Low-emission pozzolanic cement CEM IV/B(V) 42.5 N	Primary binder providing strength development	Reduced CO_2_ footprint (up to 40%) and incorporation of recycled raw materials
Fine aggregate	Quartz sand	Skeleton formation and dimensional stability	Reference natural aggregate
Recycled mineral component	Ground demolition brick (GB)	Partial replacement of natural mineral constituents	Valorization of construction and demolition waste
Recycled mineral component	Fly ash (FA)	Pozzolanic reaction and matrix densification	Utilization of industrial by-product and reduced clinker consumption
Recycled mineral component	Coal slag (SL)	Partial replacement of natural mineral materials	Reuse of industrial waste and reduction in landfill disposal
Reinforcement	Glass fibers (GF)	Improvement of tensile behavior and crack resistance	Enhanced durability and service life
Reinforcement	Merino wool fibers (MF) (post-industrial waste)	Crack control and reinforcement of the cellular matrix	Valorization of textile waste within circular economy principles
Foaming agent	Synthetic foaming agent	Generation of stable cellular structure and density reduction	Lower material consumption due to lightweight structure
Mixing water	Tap water	Hydration and mixture preparation	—

**Table 3:** Printing parameters.

Parameter	Description
3D printer	Gantry-type 3D printer
Material delivery system	20 L hopper with screw feeder
Nozzle diameter	20 mm
Layer height	8 mm
Printed filament width	20 mm
Printing speed	40 mm/s
Printing environment	22 ± 2 °C; 55 ± 5% relative humidity

**Table 4:** Characteristics of the investigated mixtures and experimental protocol (exposure condition: R—reference, A—atmospheric-aged, and FT—freeze–thaw). The checkmark (✓) indicates the sample (exposure condition) subjected to the respective test specified in the table.

Mixture	Binder	Fibers	TG-FTIR	Leaching	Microbiological
M1	Cement	0.3 GF + 0.2 MF	–	–	✓ (A)
M3	Cement	0.25 GF + 0.25 MF	✓ (R, A, FT)	✓ (A)	✓ (A)
M4	Alkali-activated	0.3 GF + 0.2 MF	–	–	✓
M6	Alkali-activated	0.25 GF + 0.25 MF	✓ (R, A, FT)	✓ (A)	✓ (A)

**Table 5:** Experimental conditions for specimen preparation, environmental exposure, and freeze–thaw testing.

Parameter	Description
Reference specimens	Stored under laboratory conditions (22 ± 2 °C; 55 ± 3% RH) throughout the entire experimental period.
Atmospheric exposure	Outdoor exposure under natural weather conditions for 6 months (November–March) in Kraków, Poland (50.057° N, 20.041° E).
Environmental monitoring	Air temperature, relative humidity, and UV index were continuously monitored using a local weather station (LSK_meteo, Kraków, Poland) equipped with a VEVOR 7-in-1 wireless meteorological system (VEVOR, Shanghai, China).
Specimen orientation	Test specimens were randomly extracted from different locations of the printed wall elements while maintaining the same orientation relative to the printing direction.
Freeze–thaw equipment	LabEvent LC/34/40/5 climatic chamber (Weiss Technik, Reiskirchen, Germany).
Temperature range	temperature range
Freeze–thaw cycle	Cooling to −40 °C (150 min), holding for 30 min, heating to 80 °C (150 min), followed by holding for 30 min.
Cycle duration	360 min.
Number of cycles	120 complete freeze–thaw cycles over 30 days, corresponding to 240 transitions through 0 °C.
Experimental replicates	At least six independent analyses were performed for each experimental variant to ensure the reproducibility of the results.

**Table 6:** Characteristic thermal decomposition parameters obtained from TG-DTG analysis for the fiber-reinforced foamed composites subjected to different conditioning regimes (R—reference, A—atmospheric-aged, and FT—freeze–thaw). Values represent the mean of three independent measurements.

Mixture	Peak I (°C)	Δm_1_ (%)	Peak II (°C)	Δm_2_ (%)	Peak III (°C)	Δm_3_ (%)	Residual Mass (%)
2-M3 R	124.6	1.25	543.7	2.51	756.2	7.49	88.76
2-M3 A	125.1	1.73	—	—	757.2	9.62	88.64
2-M3 FT	124.9	1.43	544.0	2.52	756.8	7.49	88.56
4-M6 R	111.3	1.46	554.6	6.48	720.4	1.89	90.17
4-M6 A	102.3	2.81	534.3	2.80	732.7	3.10	89.31
4-M6 FT	118.8	2.18	556.2	5.53	731.6	2.38	89.92

**Table 7:** Concentrations of selected organic contaminants and total organic carbon (TOC) of the investigated fiber-reinforced foamed composites in non-activated and activated conditions.

Parameter	Non-Activated	Activated
pH	11.00	11.40
TOC (%)	0.60	0.50
BTEX (µg/kg)	41.00	35.00
PAH (mg/kg)	<0.85	<0.85
PCB (mg/kg)	<0.007	<0.007
Mineral oil hydrocarbons (C10–C40) (mg/kg)	24.00	18.00

**Table 8:** Leaching characteristics of the investigated composites determined according to PN EN 12457-3, together with analytical limits and waste acceptance criteria.

Parameter	Unit	Non-Activated	Activated	LOD	LOQ	Inert Waste	Non-Hazardous Waste	Hazardous Waste
Arsenic (As)	mg/kg	<0.020	1.200	0.010	0.0010	0.5	2	25
Barium (Ba)	mg/kg	0.170	0.037	0.710	—	20	100	300
Cadmium (Cd)	mg/kg	<0.001	<0.001	0.001	—	0.04	1	5
Chromium (Cr)	mg/kg	3.600	4.000	0.004	0.0004	0.5	10	70
Copper (Cu)	mg/kg	0.850	4.500	0.007	0.0007	2	50	100
Mercury (Hg)	mg/kg	<0.007	<0.007	0.007	—	0.01	0.2	2
Molybdenum (Mo)	mg/kg	0.390	0.910	0.180	—	0.5	10	30
Nickel (Ni)	mg/kg	0.120	0.810	0.060	—	0.4	10	40
Lead (Pb)	mg/kg	<0.017	<0.017	0.017	—	0.5	10	50
Antimony (Sb)	mg/kg	0.073	0.260	0.017	0.0017	0.06	0.7	5
Selenium (Se)	mg/kg	<0.050	0.350	0.050	—	0.1	0.5	7
Zinc (Zn)	mg/kg	0.026	0.026	0.026	—	4	50	200
Chloride (Cl^−^)	mg/kg	240.000	240.000	1.500	—	800	15,000	25,000
Fluoride (F^−^)	mg/kg	2.300	12.000	0.500	—	10	150	500
Sulfate (SO_4_^2−^)	mg/kg	2700.000	4700.000	0.450	0.000045	1000	20,000	50,000
Total dissolved solids (TDS)	mg/kg	3700.000	18,000.000	60.000	—	4000	60,000	100,000
Phenol index	mg/kg	<0.100	<0.100	<0.100	—	1	–	–
Dissolved organic carbon (DOC)	mg/kg	218.000	509.000	1.000	—	500	800	1000

**Table 9:** Semi-quantitative (visual) rating scale for the assessment of fungal growth emerging from the specimens.

Rating	Degree of Fungal Growth on the Sample Surface
0	No visible fungal growth
1	Fungal mycelium presence (<10% of the surface)
2	Slight fungal growth (10–30% of the surface)
3	Moderate fungal growth (30–60% of the surface)
4	Extensive fungal growth (60–90% of the surface)
5	Very extensive fungal growth (>90% of the surface or complete surface coverage)

**Table 10:** Semi-quantitative ratings of fungal growth emerging from the specimens after 28 days, based on four independent replicates (plates) for each variant.

Mixture	3% Sucrose Medium	6% Sucrose Medium
M1	2	1
M3	5	5
M4	0	0
M6	0	0

## Data Availability

The original contributions presented in this study are included in the article. Further inquiries can be directed to the corresponding author.
